# The effects of prenatal metformin on obesogenic diet-induced alterations in maternal and fetal fatty acid metabolism

**DOI:** 10.1186/s12986-016-0115-9

**Published:** 2016-08-22

**Authors:** Kemoy Harris, Neeraj Desai, Madhu Gupta, Xiangying Xue, Prodyot K. Chatterjee, Burton Rochelson, Christine N. Metz

**Affiliations:** 1 Hofstra Northwell School of Medicine, Department of OB/GYN, Division of Maternal-Fetal Medicine, Manhasset, NY USA; 2 Elmezzi Graduate School of Molecular Medicine, Manhasset, NY USA; 3 Present Address: Jamaica Hospital Medical Center, Medisys Health Network, Jamaica, NY USA; 4 Winnie Palmer Hospital-Orlando Health, Orlando, FL USA; 5 The Feinstein Institute for Medical Research, The Center for Biomedical Sciences, Northwell Health, 350 Community Drive, Manhasset, NY 11030 USA

**Keywords:** Fatty acid metabolism, Fetal programming, Metabolic syndrome, Pregnancy

## Abstract

**Background:**

Maternal obesity may program the fetus and increase the susceptibility of the offspring to adult diseases. Metformin crosses the placenta and has been associated with decreased inflammation and reversal of fatty liver in obese leptin-deficient mice. We investigated the effects of metformin on maternal and fetal lipid metabolism and hepatic inflammation using a rat model of diet-induced obesity during pregnancy.

**Methods:**

Female Wistar rats (6–7 weeks old) were fed normal or high calorie diets for 5 weeks. After mating with normal-diet fed males, half of the high calorie-fed dams received metformin (300 mg/kg, daily); dams (8 per group) continued diets through gestational day 19. Maternal and fetal livers and fetal brains were analyzed for fatty acids and for fatty acid metabolism-related gene expression. Data were analyzed by ANOVA followed by Dunnett’s post hoc testing.

**Results:**

When compared to control-lean maternal livers, obesogenic-diet-exposed maternal livers showed significantly higher saturated fatty acids (14:0 and 16:0) and monounsaturated fatty acids (16:1n7 and 18:1n9) and lower polyunsaturated (18:2n6 and 20:4n6 [arachidonic acid]) and anti-inflammatory n3 polyunsaturated fatty acids (18:3n3 and 22:6n3 [docosahexaenoic acid]) (*p* < 0.05). Metformin did not affect diet-induced changes in maternal livers. Fetal livers exposed to the high calorie diet showed significantly increased saturated fatty acids (18:0) and monounsaturated fatty acids (18:1n9 and 18:1n7) and decreased polyunsaturated fatty acids (18:2n6, 20:4n6 and 22:6n3) and anti-inflammatory n3 polyunsaturated fatty acids, along with increased gene expression of fatty acid metabolism markers (*Fasn*, *D5d*, *D6d*, *Scd1*, *Lxrα*). Metformin significantly attenuated diet-induced inflammation and 18:1n9 and 22:6n3 in fetal livers, as well as n3 fatty acids (*p* < 0.05). Prenatal obesogenic diet exposure significantly increased fetal liver IFNγ levels (*p* < 0.05), which was reversed by maternal metformin treatment (*p* < 0.05).

**Conclusions:**

Consumption of a high calorie diet significantly affected maternal and fetal fatty acid metabolism. It reduced anti-inflammatory polyunsaturated fatty acids in maternal and fetal livers, altered gene expression of fatty acid metabolism markers, and induced inflammation in the fetal livers. Prenatal metformin attenuated some diet-induced fatty acid changes and inflammation in the fetal livers without affecting maternal livers, suggesting that maternal metformin may impact fetal/neonatal fatty acid/lipid metabolism.

**Electronic supplementary material:**

The online version of this article (doi:10.1186/s12986-016-0115-9) contains supplementary material, which is available to authorized users.

## Background

The prevalence of obesity and diabetes has increased in the US and globally [1–3]. Data from 2013 to 2014 reveals that over 40 % of women in the US are obese (BMI ≥ 30) and over 9 % are morbidly obese (BMI ≥ 40) – representing a linear increase since 2005 [4, 5]. Pregnancy itself is characterized by increased insulin resistance, which is more severe in gestational diabetes mellitus (GDM). Maternal obesity and GDM, along with their accompanying metabolic/lipid, vascular, and inflammatory changes [6] are associated with an increased risk of poor pregnancy outcomes [7–9], as well as an increased risk of type 2 diabetes [10, 11] and cardiovascular disease in the future [12, 13]. In addition, maternal obesity and aberrant glucose and lipid metabolism may program the fetus for hepatic lipid dysfunction and may increase the susceptibility of the offspring to adult diseases/conditions, including metabolic syndrome-like phenotype and poor vascular health [14–17].

In the US, insulin has been considered the standard therapy for GDM [18]. Metformin, a biguanide drug that promotes glucose uptake and inhibits liver gluconeogenesis, is increasingly being used during pregnancy, but is not approved by the US Food and Drug Administration for use in GDM. In the absence of pregnancy, evidence for improved lipid metabolism by metformin has been accumulating [19, 20]. In fact, metformin treatment reversed fatty liver disease in obese leptin-deficient mice [21]. Because metformin crosses the placenta [22, 23] and thus, could impact both maternal and fetal metabolism, we investigated the effects of prenatal metformin administration on maternal and fetal lipid metabolism and hepatic inflammation using a rat model of diet-induced obesity and metabolic syndrome during pregnancy.

## Methods

### Experimental animals

The Institutional Animal Care and Use Committee (IACUC) approved all animal studies prior to animal experimentation (#2010-031). All animal experimentation was performed in accordance with the Public Health Service (PHS) Policy on Humane Care and Use of Laboratory Animals and euthanasia complied with the American Veterinary Medical Association (AVMA) Panel on Euthanasia. Female Wistar rats (6–7 weeks old, Taconic, Germantown, NY) were acclimatized with free access to standard rat chow and water for at least 72 h. As previously described [24], rats were randomly assigned to one of two *ad libitum* diets: (1) a control rat chow or normal-fed (NORM) or (2) a high calorie diet (HCAL, consisting of 33 % ground commercial rat chow, 33 % full fat sweetened condensed milk, 7 % sucrose, and 27 % water) shown to induce GDM and metabolic syndrome in rats [24, 25]. After 5 weeks, lean and acutely obese female rats (maintained on their respective diets) were mated with normal-fed male Wistar rats (9–14 weeks old, Taconic). On gestation day 1 (GD1), one half of the dams fed the high calorie diet received metformin (300 mg/kg, p.o. daily). All dams (NORM, *n* = 8; HCAL, *n* = 8; HCAL + metformin, *n* = 8) continued their respective diets throughout gestation. On GD19 dams were euthanized by CO_2_ inhalation followed by exsanguination and livers were collected. Fetuses, delivered by cesarean section were euthanized by decapitation and fetal livers and brains were collected. Maternal livers, fetal livers and fetal brains were flash frozen in liquid nitrogen and stored at −80 °C.

### Lipid and fatty acid (FA) profiling

Lipids were extracted from the maternal livers and fetal livers and brains according to the method of Folch–Lees [26]. Individual fatty acids (FAs) of the triglyceride fractions (maternal and fetal livers) or phospholipid fractions (fetal brains) were quantified at the Lipid Core Laboratory of Vanderbilt University’s Mouse Metabolic Phenotyping Center (Grant #DK59637), as previously described [27]. Total triglyceride-associated FAs of the maternal and fetal livers were normalized to liver weights (μg/mg); FA subsets and individuals FAs were expressed as percent of total fraction (mg/100 mg triglyceride-fatty acids (maternal and fetal livers) or phospholipid fatty acids (fetal brains)).

### Expression of markers of FA metabolism

Markers of FA synthesis and metabolism in maternal and fetal livers and fetal brains were assessed by quantitative polymerase chain reaction (qPCR) methods, as previously described [28]. RNA was isolated using the RNeasy® Plus Universal Mini kit with DNase treatment (Qiagen, Foster City, CA); RNA samples had 260/280 and 260/230 ratios ≥1.9. qPCR reactions were performed (in triplicate) using rat specific primers (designed using the Roche Universal Probe Library Design Center) and synthesized by Eurofins (Huntsville, AL) for rat desaturase and elongase-related genes: *D5d*, *D6d*, *Scd1*, *Elovl1*, *Elovl2*, *Elovl5*, and *Elovl6* and other genes involved in FA synthesis: *Chrebp* (also known as *Mlxipl*), *Lxra*, *Lxrb*, *Srebf1*, *and Fasn*, using the Eurogentec One Step RT qPCR mastermix (AnaSpec, Fremont, CA), 100 ng RNA, Roche Universal Library probes (Indianapolis, IN) and the Roche 480 Light Cycler, as previously described [29]. See Table 1 for qPCR primers and probes. Changes in mRNA expression (corrected using rat *Hprt1* as the housekeeping gene) were calculated using the comparative Ct (ΔΔCt) method [30]. Data are presented as relative mRNA expression with the normal-fed group set to 1.0.

**Table 1:** qPCR primers used to assess gene expression of markers of lipid/fatty acid metabolism

Gene	Primer	Sequence 5′ ^_^ 3′	Accession number^a^ (Probe number)
*Chrebp*	Forward Reverse	AATCCCAGCCCCTACACC CTGGGAGGAGCCAATGTG	NM_133552.1 (10)
*D5d*	Forward Reverse	GAACTCTCTTCTGATTGGAGAGCTA CCGGAATTCATCAGTGAGC	AB052085.1 (26)
*D6d*	Forward Reverse	AATTTCCAGATTGAGCACCAC AGTGGGGCAATCTTGTGC	NM_031344.2 (60)
*D9d*	Forward Reverse	GAAGCGAGCAACCGACAG GGTGGTCGTGTAGGAACTGG	NM_139192.2 (125)
*Elovl2*	Forward Reverse	AACCTCGGAATCACACTTCTTT TCCCAGCTGGAGAGAACG	NM_001109118.1 (22)
*Elovl5*	Forward Reverse	TCGATGCGTCACTCAGTACC CCTTTGACTCGTGTGTCTCG	NM_134382.1 (122)
*Elovl6*	Forward Reverse	ATGGATGCAGGAAAACTGGA GCCCGCTTGTTCATCAGA	NM_134383.2 (119)
*Fasn*	Forward Reverse	GGCCACCTCAGTCCTGTTAT AGGGTCCAGCTAGAGGGTACA	NM_017332.1 (6)
*Lxrα*	Forward Reverse	CAGGAAGAGATGTCCTTGTGG TCTTCCACAACTCCGTTGC	NM_031627.2 (2)
*Lxrβ*	Forward Reverse	AGCTCTGCCTACATCGTGGT GACCCTTCTTCCGCTTGC	NM_031626.1 (106)
*Srebf*	Forward Reverse	ACAAGATTGTGGAGCTCAAGG TGCGCAAGACAGCAGATTTA	NM_001276707.1 (77)

Forward and reverse primers for rat genes with GenBank Accession numbers and specific Roche Universal Probe numbers used for assessing mRNA expression in rat tissues by qPCR

^a^National Center for Biotechnology Information (NCBI) EntrezGene (http://www.ncbi.nlm.nih.gov/gene)

### Evaluation of cytokines and chemokines in maternal and fetal livers

Maternal and fetal livers were analyzed for multiple cytokines using the rat 9-plex pro-inflammatory panel assay kit (Meso Scale Diagnostics [MSD], Rockville, MD), as previously described [31]. Liver homogenates were assayed for cytokines (IFNγ, IL-1β, IL-4, IL-5, IL-6, IL-10, IL-13, and TNFα) and chemokines (CXCL1, CXC-motif ligand 1) using the MSD multiplex platform. The raw data were measured as electrochemiluminescence signals with the MSD Sector Imager 2400 plate reader (Meso Scale Diagnostics, Rockville, MD) and analyzed using the Discovery Workbench 3.0 software (MSD). The lower limits of detection for the analytes in this assay were ≤2 pg/ml, except for: IL-1β (7 pg/ml), IL-5 and IL-6 (14 pg/ml), and IL-10 (20 pg/ml). The *R*^2^ value for each standard curve was between 0.99 and 1.0. Samples being compared were run on the same plate and the % coefficient of variation of control replicates run on the same plate was between 2.3 % (TNFα) and 5.7 % (IFNγ). Liver cytokine data were corrected for protein concentration (Bio-Rad protein assay, Bio-Rad, Hercules, CA) and expressed as pg/g.

### Western blotting

ChREBP and SREBP-1 protein expression in maternal and fetal livers was assessed by western blotting. Liver tissues (100 mg) were homogenized as described above. Proteins (40 μg/lane, determined by BioRad protein assay) were separated by electrophoresis using Nu-PAGE® gels (Life Technologies, Grand Island, NY) and transferred to PVDF membranes (Millipore). The blots were probed with anti-SREBP-1 (Santa Cruz Biotechnology, Dallas TX), anti-ChREBP (Novus Biologicals, Littleton, CO), and anti-GAPDH (Cell Signaling Technology, Danvers, MA, USA) antibodies, followed by near infrared-fluorescently labeled secondary antibodies (LI-COR, diluted 1:15,000), and revealed using the Odyssey infrared imaging system (LI-COR Biosciences), as previously described [28, 32]. Band densities (SREBP-1: GAPDH and ChREBP:GAPDH) were assessed using Image J software (NIH).

### Statistics

Data were analyzed using one-way ANOVAs for multiple comparisons followed by Dunnett’s post-hoc testing using GraphPad Prism 5.03 (GraphPad Software, San Diego, CA). *P* values <0.05 were considered significant.

## Results

### High calorie diet alters maternal liver lipid profiles: no effect of metformin

**Fig. 1:**
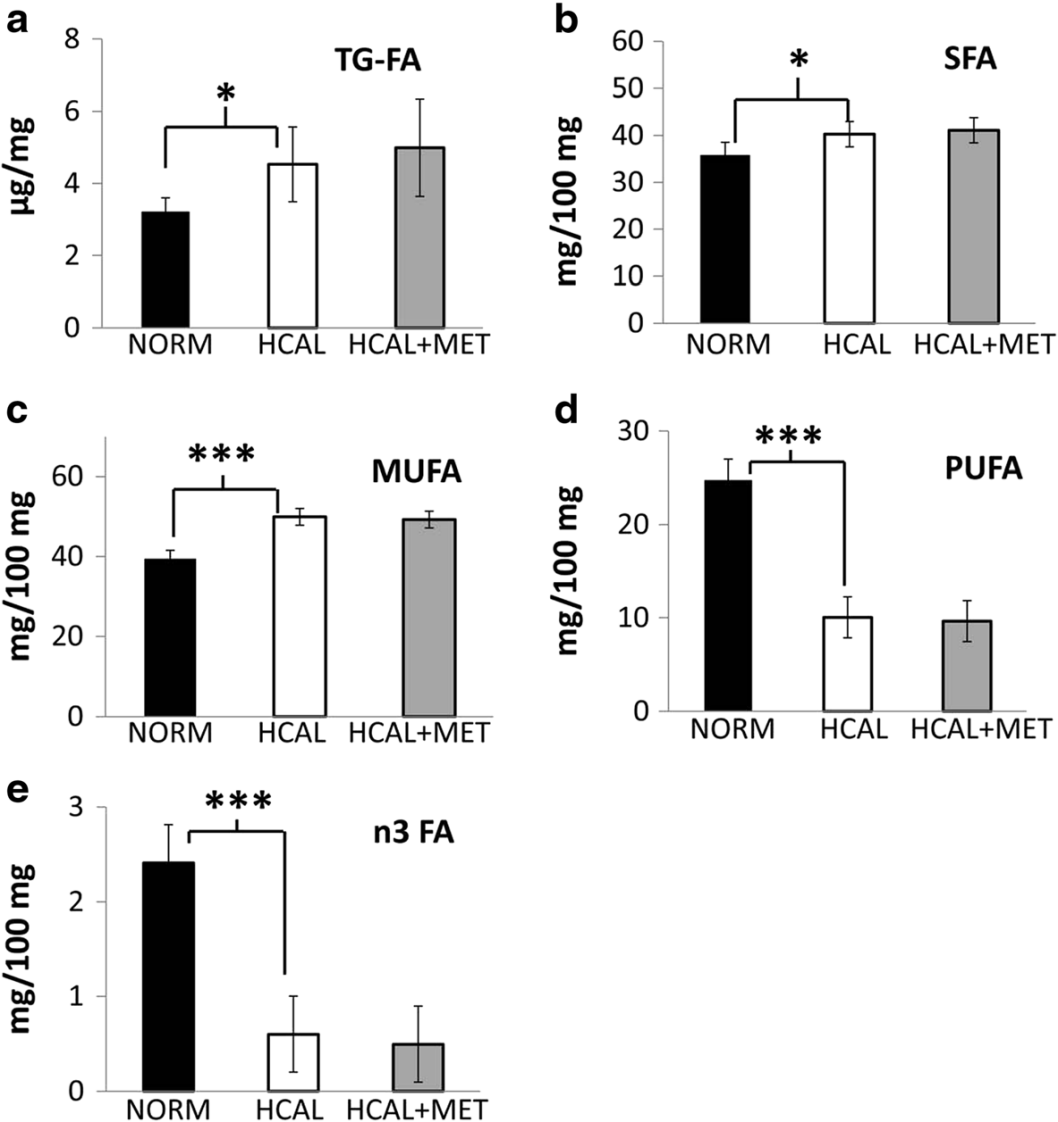
HCAL diet increases triglyceride deposition in the maternal liver and alters maternal liver lipid profiles. No effect of metformin. Dams were fed a normal (NORM) or a high calorie (HCAL) diet prior to and during pregnancy ± metformin (MET), as described in the Methods. Maternal liver total triglyceride-associated FA (TG-FA) concentrations were determined on GD19 (**a**). Saturated fatty acids (SFA, **b**), monounsaturated FA (MUFA, **c**), polyunsaturated FA (PUFA, **d**), and n3 fatty acids (n3FA, **e**) profiles of the maternal livers were determined on GD19. Data are shown as mean ± SD. *p < 0.05; **p < 0.01; ***p < 0.001

Livers obtained from the HCAL-fed dams showed a 40 % increase in total triglyceride-associated FAs vs. control dam livers, irrespective of metformin treatment (Fig. 1a, *p* < 0.05). Furthermore, maternal livers showed numerous HCAL-diet related changes in their FA profiles; HCAL-exposed livers had significantly higher saturated fatty acids (SFAs) (Fig. 1b, *p* < 0.05) and monounsaturated fatty acids (MUFAs) (Fig. 1c, *p* < 0.001) and lower polyunsaturated fatty acids (PUFAs) (Fig. 1d, *p* < 0.001), as well as a 3-fold lower expression of anti-inflammatory n3 FAs compared to control-fed dams (Fig. 1e, *p* < 0.001). More specifically, maternal HCAL livers had significantly higher 14:0, 16:0, 16:1n7, and 18:1n9 FAs and significantly lower 18:2n6, 18:3n3, 20:4n6 (arachidonic acid, AA) and 22:6n3 (docosahexaenoic acid, DHA) FAs, when compared to livers obtained from control-fed dams (Table 2). Maternal metformin treatment did not affect HCAL-diet-induced triglyceride-FA levels or FA-related changes in the maternal livers (Fig. 1a-e and Table 2).

**Table 2:** HCAL diet affects maternal liver fatty acid profiles. No effect of metformin

Fatty acid		NORM mean (±SD)	HCAL mean (±SD)	HCAL + MET mean (±SD)
SFA	14.0	0.6 (±0.14)	1.3 (±0.24)^a**^	1.2 (±0.20)
	16.0	28.5 (±1.90)	32.7 (±2.35)^a*^	33.4 (±2.25)
	18.0	6.7 (±0.44)	6.3 (±1.89)	6.5 (±0.60)
MUFA	16.1n7	2.1 (±0.47)	4.2 (±0.89)^a*^	3.7 (±0.47)
	18.1n9	33.2 (±1.43)	41.4 (±1.04)^a*^	41.3 (±1.79)
	18.1n7	4.1 (±0.29)	4.4 (±0.44)	4.2 (±0.74)
PUFA	18.2n6	19.5 (±2.60)	8.3 (±1.41)^a*^	8.1 (±1.10)
	18.3n3	0.8 (±0.10)	0.3 (±0.19)^a*^	0.2 (±0.21)
	20.4n6	2.6 (±0.74)	1.1 (±0.34)^a*^	1.1 (±0.39)
	22.6n3	1.6 (±0.63)	0.3 (±0.23)^a*^	0.2 (±0.22)

*NORM* normal diet, *HCAL* high calorie diet, *MET* metformin, *SD* standard deviation, *SFA* saturated fatty acids, *MUFA* monounsaturated fatty acids, *PUFA* polyunsaturated fatty acids

^a^NORM vs. HCAL; *p < 0.05, **p < 0.01

Data are expressed as mean ± SD (mg/100 mg)

### Maternal obesogenic diet affects markers of lipid metabolism in maternal livers

**Fig. 2:**
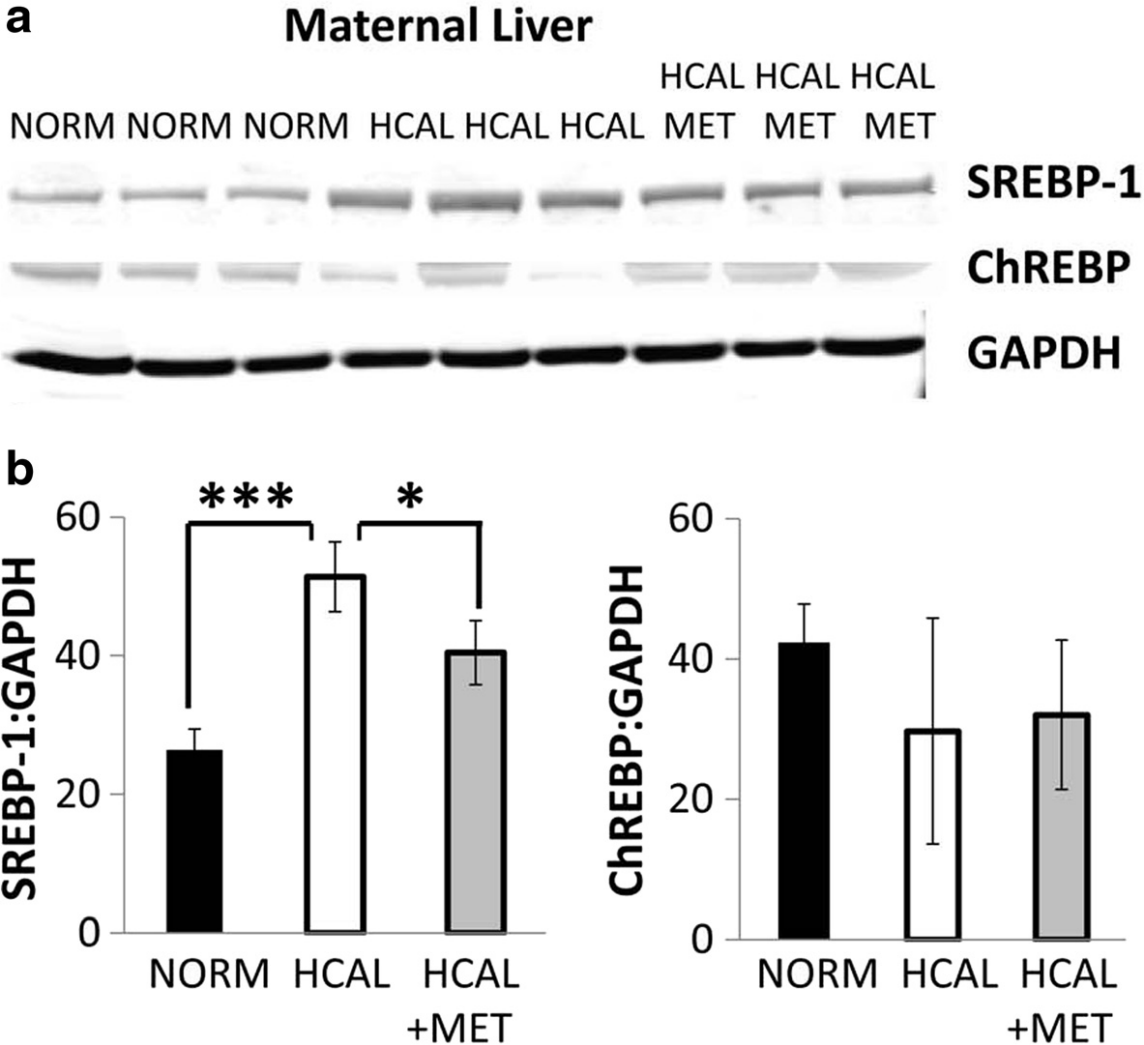
*SREBP-1 protein in the maternal liver is increased by the HCAL diet*. Maternal livers obtained from dams after feeding normal (NORM) or high calorie (HCAL) diets (±metformin, MET) were examined on GD19 for SREBP-1 and ChREBP protein expression by western blotting (**a**). Quantitation of SREBP-1 and ChREBP bands normalized for GAPDH expression (SREBP-1:GAPDH and ChREBP:GAPDH) is shown in **b**. Data are shown as mean ± SD. *p < 0.05; ***p < 0.001

*Srebf1* mRNA expression was significantly induced in the maternal livers following HCAL diet exposure (Table 3, *p* < 0.001). However, maternal metformin administration did not alter HCAL-induced *Srebf1* mRNA expression in the maternal livers (Table 3). There were no significant changes in the mRNA expression of other markers of lipid synthesis/metabolism analyzed (*Chrebp*, Lxrα/β, *Srebf1*, and *Fasn*, as well as various elongases (*Elovl2*, *5*, or *6*) and desaturases (*D5d*, *D6d*, *D9d [Scd1]*)) in the maternal livers following the HCAL diet (±metformin) (Table 3). Next, we confirmed the increased expression of SREBP-1 protein in maternal livers following HCAL diet exposure when compared to controls (Fig. 2a and b, *p* < 0.001); the increased SREBP-1 protein expression was partially attenuated by metformin administration (Fig. 2a and b, *p* < 0.05). By contrast, maternal liver ChREBP protein expression was unaffected by HCAL-diet exposure (± metformin) (Fig. 2a and b).

**Table 3:** The effects of high calorie diet (± metformin) on gene expression in maternal and fetal livers

Maternal livers				Fetal Livers		
Gene	NORM mean (±SD)	HCAL mean (±SD)	HCAL + MET mean (±SD)	NORM mean (±SD)	HCAL mean (±SD)	HCAL + MET mean (±SD)
*Chrebp*	1.0 (±0.33)	0.9 (±0.19)	1.1 (±0.41)	1.0 (±0.25)	1.1 (±0.34)	0.9 (±0.46)
*D5d*	1.0 (±0.36)	1.2 (±0.23)	1.4 (±0.27)	1.0 (±0.34)	1.5 (±0.44)^a*^	1.4 (±0.34)
*D6d*	1.0 (±0.49)	1.1 (±0.33)	1.5 (±0.26)	1.0 (±0.31)	1.5 (±0.23)^a***^	1.6 (±0.26)
*D9d (Scd1)*	1.0 (±0.68)	1.2 (±0.35)	1.3 (±0.33)	1.0 (±0.33)	1.4 (±0.37)^a*^	1.2 (±0.28)
*Elovl2*	1.0 (±0.39)	0.9 (±0.24)	0.8 (±0.13)	1.0 (±0.23)	1.1 (±0.30)	1.0 (±0.32)
*Elovl5*	1.0 (±0.14)	1.0 (±0.24)	1.1 (±0.06)	1.0 (±0.12)	1.5 (±0.86)	1.1 (±0.18)
*Elovl6*	1.0 (±0.37)	0.8 (±0.26)	1.0 (±0.43)	1.0 (±0.15)	1.1 (±0.16)	1.3 (±0.25)
*Fasn*	1.0 (±0.46)	1.0 (±0.47)	1.1 (±0.39)	1.0 (±0.18)	1.5 (±0.28)^a***^	1.6 (±0.37)
*Lxrα*	1.0 (±0.13)	1.0 (±0.09)	1.1 (±0.14)	1.0 (±0.13)	1.2 (±0.30)^a**^	1.3 (±0.30)
*Lxrβ*	1.0 (±0.42)	1.0 (±0.11)	1.0 (±0.13)	1.0 (±0.13)	1.0 (±0.14)	1.0 (±0.13)
*Srebf1*	1.0 (±0.29)	1.8 (±0.36)^a***^	1.7 (±0.38)	1.0 (±0.23)	1.3 (±0.40)	1.2 (±0.19)

*NORM* normal diet, *HCAL* high calorie diet, *MET* metformin, *SD* standard deviation

^a^NORM vs. HCAL; *p = <0.05; **p < 0.01, ***p < 0.001

Data are presented as relative mRNA expression (mean ± SD), with the normal-fed group set to 1.0

### HCAL diet prior to and during pregnancy did not promote cytokine production in maternal livers

Based on the link between hepatic liver lipid accumulation and inflammation, we assessed maternal livers obtained from control-fed and HCAL-fed (±metformin) dams for numerous cytokines. We found no effect of the HCAL diet (with or without metformin) on localized hepatic inflammation, as determined by measuring IL-1β, IL-4, IL-5, IL-6, IL-10, IL-13, IFNγ, TNFα, and CXCL1 concentrations in the maternal livers (Additional file 1: Table S1).

### Fetal exposure to maternal obesity significantly alters fetal hepatic FA profile; metformin reverses some of these effects

**Fig. 3:**
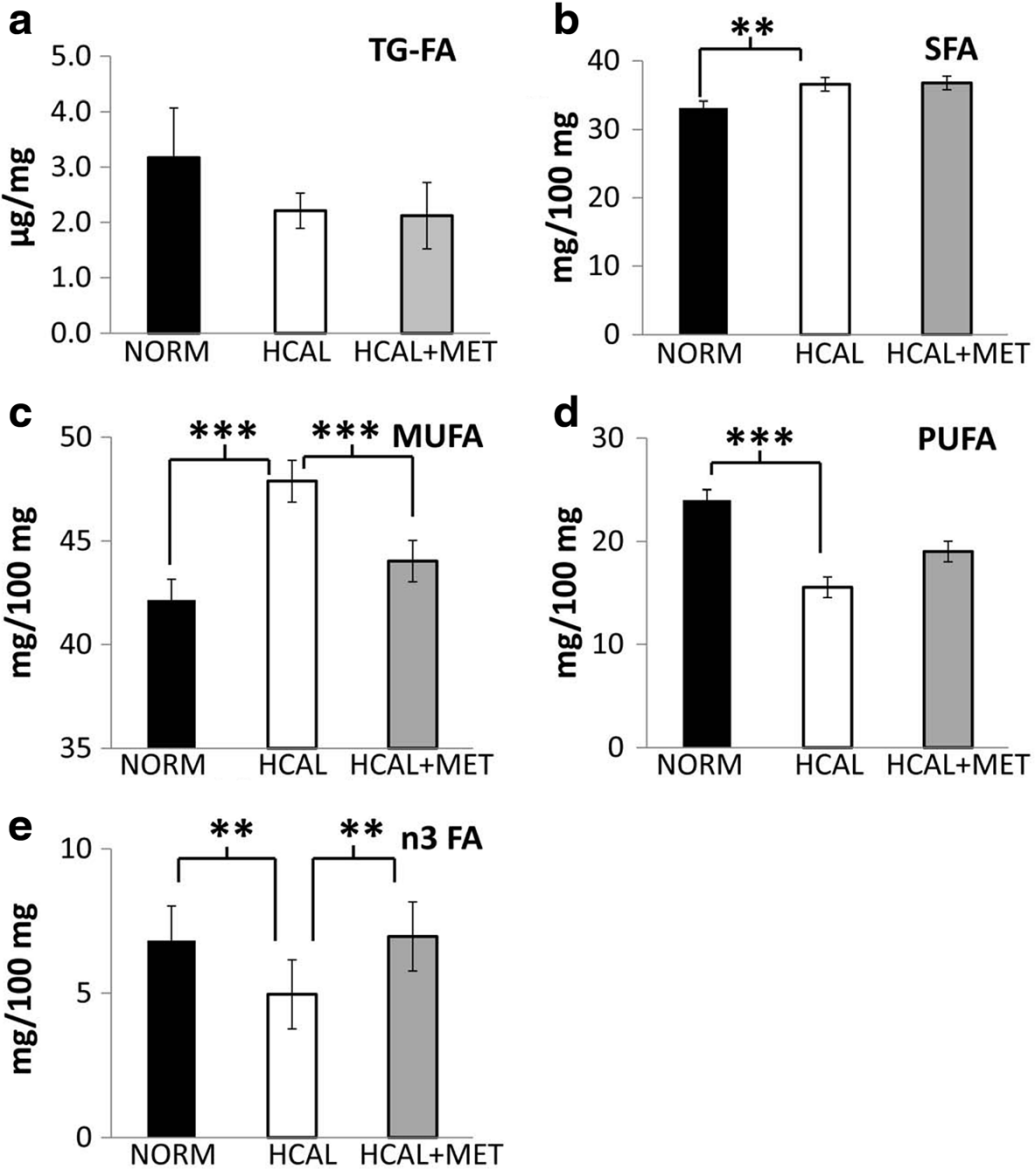
*HCAL diet exposure alters fetal liver fatty acid profiles. Modifications by in utero metformin exposure*. Fetuses were exposed to either a normal (NORM) or a high calorie (HCAL) maternal diet (±metformin, MET), as described in the Methods. Fetal liver total triglyceride-associated fatty acid (TG-FA) concentrations (**a**), saturated fatty acids (SFA, **b**), monounsaturated FA (MUFA, **c**), polyunsaturated FA (PUFA, **d**), and n3 FA (**e**) profiles of the fetal livers were determined on embryonic day 19. Data are shown as mean ± SD.*p < 0.05; ***p < 0.001

Although *in utero* exposure to the HCAL diet did not significantly change the total triglyceride-associated FA content of the fetal livers on embryonic day 19 (Fig. 3a), it significantly increased SFAs (Fig. 3b, *p* < 0.01) and MUFAs (Fig. 3c, *p* < 0.001), and significantly decreased PUFAs (Fig. 3d, *p* < 0.001), as well as anti-inflammatory n3 FAs when compared to control fetal livers (Fig. 3e, *p* < 0.001). More specifically, livers obtained from HCAL diet-exposed fetuses showed significantly increased 18:0, 18:1n9, and 18:1n7 and significantly reduced 18:2n6, 20:4n6 (AA), and 22:6n3 (DHA) (Table 4). While no changes in maternal hepatic FA profiles were observed following metformin administration (Fig. 1b–e and Table 2), significant changes in diet-induced FA changes in the fetal livers were observed following maternal metformin treatment. HCAL-induced overall MUFAs (Fig. 3c, *p* < 0.001), specifically 18:1n9, were significantly attenuated by maternal metformin administration (Table 4, *p* < 0.01), while HCAL-induced 18:1n7 was only slightly reduced (not significant) (Table 4). Maternal metformin blocked HCAL-reduced 22:6n3 (DHA) in fetal livers following HCAL-exposure (Table 4, *p* < 0.05). Similarly, HCAL-suppressed n3 FA levels in the fetal livers were reversed by maternal metformin treatment (Fig. 3e, *p* < 0.001).

**Table 4:** HCAL diet (±metformin) exposure alters FA profiles of fetal livers

Fatty acid		NORM mean (±SD)	HCAL mean (±SD)	HCAL + MET mean (±SD)
SFA	14.0	2.1 (±0.18)	2.2 (±0.27)	2.1 (±0.23)
	16.0	23.9 (±0.96)	25.6 (±1.57)	25.4 (±1.94)
	18.0	7.18 (±0.73)	8.8 (±0.87)^a***^	9.2 (±0.76)
MUFA	16.1n7	4.3 (±0.45)	4.6 (±0.46)	3.9 (±0.29)
	18.1n9	34.2 (±1.56)	38.8 (±2.08)^a**^	35.9 (±2.26)^b**^
	18.1n7	3.6 (±0.28)	4.4 (±0.20)^a***^	4.2 (±0.17)
PUFA	18.2n6	14.4 (±2.1)	8.8 (±1.70)^a***^	10.1 (±0.92)
	18.3n6	0.5 (±0.26)	0.4 (±0.21)	0.22 (±0.28)
	20.4n6	2.2 (±0.69)	1.4 (±0.43)^a*^	1.7 (±0.31)
	22.6n3	6.8 (±1.02)	5.0 (±1.76)^a*^	7.0 (±1.21)^b*^

*NORM* normal diet, *HCAL* high calorie diet, *MET* metformin, *SD* standard deviation, *SFA* saturated fatty acids, *MUFA* monounsaturated fatty acids, *PUFA* polyunsaturated fatty acids

^a^NORM vs. HCAL, ^b^HCAL vs. HCAL + MET; *p < 0.05, **p < 0.01, ***p < 0.001

Data are expressed as mean ± SD (mg/100 mg)

### In utero exposure to maternal high calorie diet alters markers of lipid metabolism in fetal livers

**Fig. 4:**
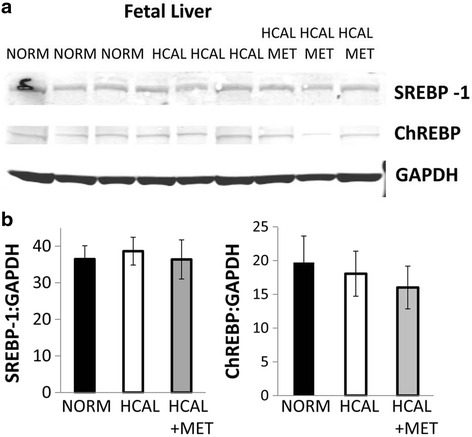
*SREBP-1 and ChREBP protein expression in the fetal livers are unaffected by exposure to the HCAL diet. No effect of metformin*. Fetal livers were examined for SREBP-1 and ChREBP protein expression by western blotting (**a**). Quantitation of SREBP-1 and ChREBP bands normalized for GAPDH expression (SREBP-1:GAPDH and ChREBP:GAPDH) are shown in **b**

Fetal exposure to the maternal HCAL-diet significantly increased the expression of several genes shown to be involved in fatty acid metabolism in the fetal livers, including *D5d*, *D6d*, *D9d* [*Scd1*], *Fasn*, and *Lxrα* (Table 3). *In utero* metformin exposure did not significantly attenuate these effects; however, metformin slightly (not significantly) reduced the effects of HCAL diet exposure on *D5d* and *D9d (Scd1)* mRNA expression in fetal livers when assessed on embryonic day 19 (Table 3). Western blotting of fetal livers confirmed that neither SREBP-1 nor ChREBP protein expression was affected by HCAL diet (with or without metformin) (Fig. 4a–b).

### In utero exposure to maternal obesogenic diet significantly affects fetal brain FA profiles

**Fig. 5:**
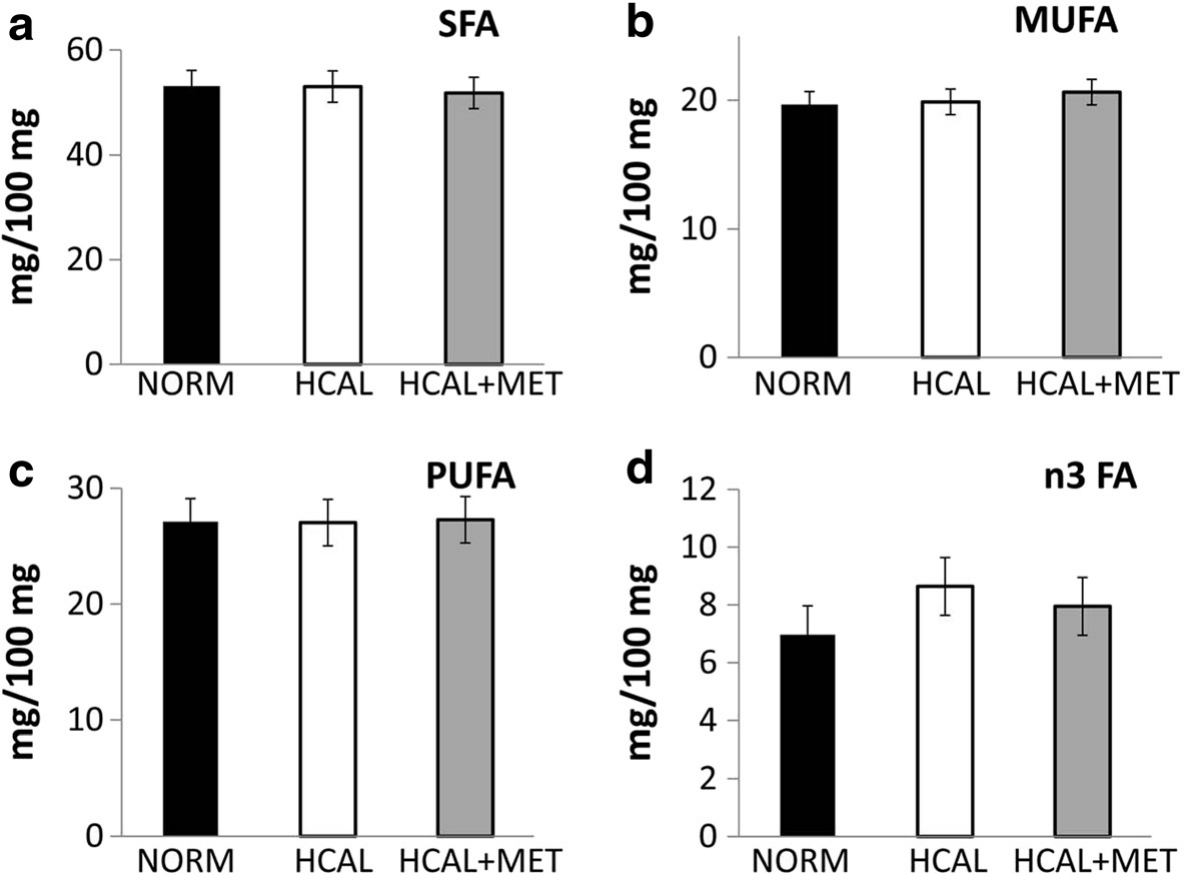
*Effect of HCAL diet exposure on fetal brain fatty acid profiles. No effect of metformin*. Fetuses were exposed to either a normal (NORM) or a high calorie (HCAL) maternal diet (±metformin, MET), as described in the Methods. The saturated fatty acids (SFA, **a**), monounsaturated FA (MUFA, **b**), polyunsaturated FA (PUFA, **c**), and n3 FA (**d**) profiles of the fetal brains were determined on embryonic day 19. Data are shown as mean ± SD

While fetal brains had no significant differences in overall phospholipid-associated SFA, MUFA, PUFA, or n3 FA concentrations following HCAL-diet exposure when compared to controls (Fig. 5a–d), HCAL diet-exposed fetal brains had significantly reduced 20:4n6 (AA), 22:4n6, and 22:5n6 and significantly increased 22:6n3 (DHA) concentrations when compared to controls (Table 5). With the exception of HCAL-reduced 22:5n6 in the fetal brains, which was reversed by maternal metformin treatment (*p* < 0.05), no other changes were observed following maternal metformin administration (Table 5). Fetal exposure to the HCAL-diet reduced *D9d* (*Scd1*) mRNA expression in the fetal brains (Table 6, *p* < 0.05), but maternal metformin had no effect on *D9d (Scd1)* mRNA expression (Table 6).

**Table 5:** Effects of maternal HCAL diet (±metformin) on fetal brain FA profiles

Fatty acid		NORM mean (±SD)	HCAL mean (±SD)	HCAL + MET mean (±SD)
SFA	14.0	1.9 (±0.08)	2.0 (±0.05)	2.0 (±0.23)
	16.0	33.4 (±0.89)	33.6 (±0.93)	32.8 (±0.27)
	18.0	17.8 (±0.81)	17.5 (±0.52)	16.9 (±0.30)
MUFA	16.1n7	2.2 (±0.21)	2.2 (±0.10)	2.3 (±0.12)
	18.1n9	13.6 (±0.56)	13.9 (±0.30)	14.3 (±0.39)
	18.1n7	3.9 (±0.19)	3.9 (±0.26)	4.0 (±0.21)
PUFA	20.4n6	13.2 (±0.42)	12.5 (±0.35)^a*^	13.0 (±0.41)
	22.4n6	3.2 (±0.12)	2.9 (±0.03)^a**^	3.0 (±0.17)
	22.5n6	2.9 (±0.24)	2.3 (±0.22)^a**^	2.7 (±0.22)^b*^
	22.6n3	7.0 (±0.75)	8.6 (±1.53)^a*^	8.0 (±0.35)

*NORM* normal diet, *HCAL* high calorie diet, *MET* metformin, *SD* standard deviation, *SFA* saturated fatty acids, *MUFA* monounsaturated fatty acids, *PUFA* polyunsaturated fatty acids

^a^NORM vs. HCAL,^b^ HCAL vs. HCAL + MET; *p < 0.05, **p < 0.01

Data are expressed as mean ± SD (mg/100 mg)

**Table 6:** The effects of HCAL diet (± metformin) on gene expression in fetal brains

Gene	NORM mean (±SD)	HCAL mean (±SD)	HCAL + MET mean (±SD)
*Chrebp*	ND	ND	ND
*D5d*	1.0 (±0.14)	0.9 (±0.13)	1.0 (±0.19)
*D6d*	1.0 (±0.06)	0.9 (±0.12)	1.1 (±0.09)
*D9d (Scd1)*	1.0 (±0.06)	0.9 (±0.11)^a***^	0.8 (±0.05)
*Elovl2*	1.0 (±0.10)	1.4 (±0.42)	1.5 (±0.08)
*Elovl5*	1.0 (±0.20)	1.0 (±0.14)	1.0 (±0.10)
*Elovl6*	1.0 (±0.15)	0.9 (±0.17)	1.1 (±0.16)
*Fasn*	1.0 (±0.13)	1.0 (±0.07)	1.0 (±0.10)
*Lxrα*	1.0 (±0.01)	1.2 (±0.07)	1.4 (±0.29)
*Lxrβ*	1.0 (±0.19)	0.9 (±0.09)	0.8 (±0.22)
*Srebf1*	1.0 (±0.14)	1.1 (±0.23)	1.0 (±0.27)

*NORM* normal diet, *HCAL* high calorie diet, *MET* metformin, *SD* standard deviation

^a^NORM vs. HCAL; ***p < 0.001

Data are presented as relative mRNA expression (mean ± SD), with the normal-fed group set to 1.0

### Prenatal exposure to metformin reduces HCAL diet-induced IFNγ levels in fetal livers

**Fig. 6:**
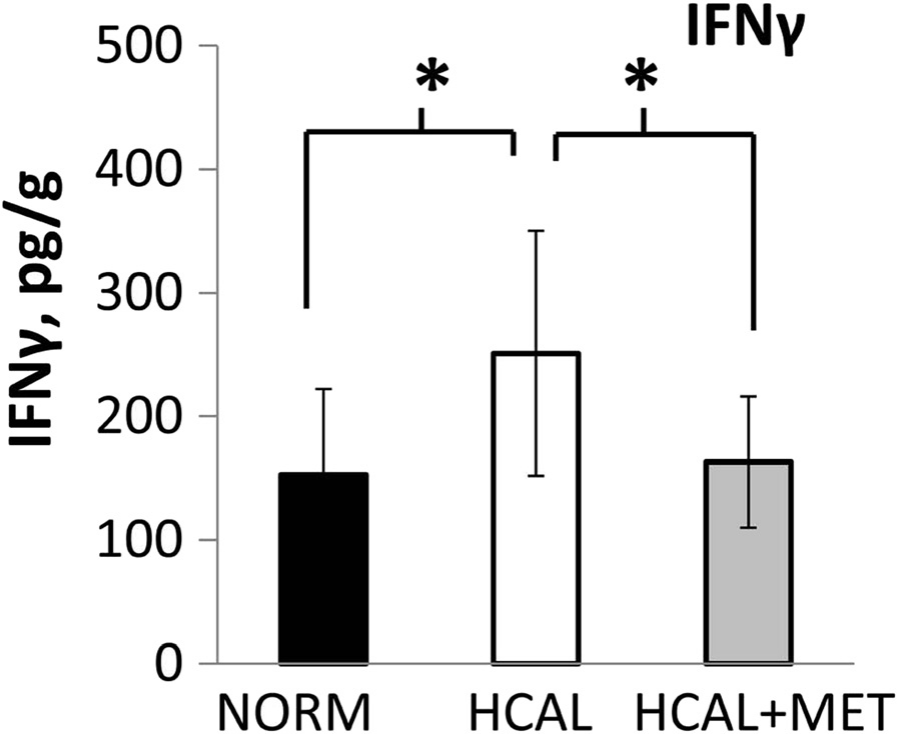
Intrauterine exposure to HCAL diet increases fetal liver IFNγ levels. Metformin attenuates enhanced IFNγ levels in fetal livers. Fetal livers were obtained following exposure to maternal normal (NORM) or high calorie (HCAL) diets (±metformin, MET) on embryonic day 19 and IFNγ levels were assessed and adjusted for fetal liver protein concentration; data are shown as mean ± SD. *p < 0.05

Although no effect of the HCAL diet was found on maternal liver inflammation, as assessed by measuring various cytokines and chemokines (see Additional file 1: Table S1), *in utero* exposure to the maternal obesogenic diet significantly increased IFNγ concentrations in the fetal livers (Fig. 6 and Additional file 1: Table S1). This effect was attenuated by prenatal metformin exposure (Fig. 6 and Additional file 1: Table S1).

## Discussion

Obesity in pregnant women is accompanied by dyslipidemia, characterized by elevated triglyceride levels [6]. Consumption of the HCAL diet enriched in saturated fats and sugar by pregnant rats significantly increased total triglycerides and increased proportion of SFAs, and MUFAs, and decreased PUFAs and n3 FAs in maternal rat livers (Fig. 1 and Table 2). The most notable changes in the maternal livers were the >2-fold decline in PUFAs (Fig. 1d) and the 3-fold decline in anti-inflammatory n3 FAs (Fig. 1e). These changes might be detrimental, as maternal PUFAs are essential for healthy fetal development, including the fetal brain [33] and because n3 FAs have been proposed to regulate fetal brain development [34] and improve insulin resistance during pregnancy [35]. Likewise, the accumulation of the end product of de novo lipogenesis, 18:1n9 (oleic acid), was significantly higher in the HCAL-exposed maternal livers when compared to control maternal livers (Table 2). These changes, indicative of liver dysfunction and enhanced de novo lipogenesis, were accompanied by elevated *Srebf* mRNA expression (almost 2-fold, Table 3) and increased SREBP-1 protein (Fig. 2a–b) in the maternal livers following the obesogenic diet. SREBP-1 serves as one of the major regulators of de novo lipogenesis/fatty acid synthesis by activating genes required for lipogenesis [36, 37]. In fact, overexpression of SREBP-1 in transgenic mice produced fatty livers via excess de novo lipogenesis [38]. Hepatic triglyceride deposition and metabolic syndrome have been strongly associated with insulin resistance [39, 40]. Both insulin and glucose are the major inducers of *Srebf* mRNA expression, and thus, these results are consistent with previous studies showing that this obesogenic diet induces GDM [25] and metabolic syndrome, as determined by increased maternal circulating insulin, leptin, and triglyceride levels [24].

Maternal obesity and metabolic syndrome can lead to adverse fetal outcomes, including structural anomalies and fetal liver lipotoxicity [41, 42]. Fetal exposure to excess maternal lipids and their metabolites are proposed to trigger signaling pathways in developing organs, including the liver, adipose, skeletal muscle, and brain, that lead to short- and long-term metabolic consequences (e.g., energy storage, cell differentiation, cell death, and inflammation) (Reviewed in [41]). We found that *in utero* HCAL exposure did not affect total triglyceride-associated fatty acids in the fetal livers, but it significantly increased the proportion of SFAs and MUFAs, decreased PUFAs and n3 FAs (Fig. 3b–e and Table 4), and elevated IFNγ in the fetal livers (Fig. 6). IFNγ, previously shown to accompany hepatic steatosis in mice [43], may directly induce endoplasmic reticulum stress, a potential contributor to the vicious cycle of obesity and chronic inflammation [35]. HCAL diet-exposed fetal livers showed enhanced *Lxra* mRNA expression (Table 3). *Lxrα* is considered the master regulator of hepatic lipogenesis; it induces *Fasn* [36, 37]. We observed enhanced *Fasn*, *D5d*, *D6d*, and *D9d* mRNA expression in the HCAL-exposed fetal livers (Table 3), consistent with previous studies showing altered FA metabolism and lipid accumulation in the fetal livers of non-human primates following exposure to a chronic maternal high fat diet [42].

Aberrant FA profiles found in the fetal brains following *in utero* HCAL-diet exposure (decreased 20:4n6, 22:4n6, and 22:5n6 (PUFAs), Table 5) were consistent with decreased PUFAs in the maternal and fetal livers (Figs. 1 and 3, Tables 2 and 4). Surprisingly, fetal brains exposed to the HCAL diet showed increased 22:6n3 (DHA) (Table 5). DHA, which is primarily obtained via transplacental transport from the maternal side, is critically important for fetal brain development [33]. Maternal liver DHA levels were more reduced by the HCAL diet when compared to those in the fetal liver. Thus, the observed increase in fetal brain DHA levels may represent a fetal compensatory mechanism to obtain DHA from the maternal compartment to protect the fetal brain despite reduced DHA availability and reduced fetal liver DHA levels.

Aberrant liver lipid metabolism and fatty liver disease are significant contributors to the morbidity and mortality associated with obesity-related diabetes [44]. Treating gestational diabetics through diet modification, exercise, and drugs (e.g., insulin and oral anti-diabetic agents, including metformin) improves maternal, fetal/neonatal, and offspring outcomes [45]. In the non-pregnant state, metformin enhances glucose regulation and lipid metabolism in obese women [19, 46] and diabetic/hyperinsulinemic rodents [20, 47]. Although it has been safely used for treating GDM in other countries since the 1970s [48], metformin has only been regularly prescribed to women with GDM in the US within recent years [49]. Similarly, treatment of GDM with metformin has recently increased in Norway, Wales, and the rest of the UK [50]. Evidence from randomized controlled trials and observational studies revealing no adverse maternal or fetal/neonatal effects in the short-term [51, 52] supports using metformin for GDM [18]. The 2013 MiG trial comparing insulin vs. metformin for treating gestational diabetics confirmed the safety of metformin in pregnancy and revealed no differences in either maternal circulating hormones/metabolites, birth weight, or neonatal anthropometric measurements between the two treatments [53]. The results of the MiG trial showed only subtle effects of metformin on maternal lipid parameters associated with cardiovascular risk and no effects on cord blood (fetal) lipids [53]. However, invasive antenatal lipid analyses, as reported herein, were not performed.

Metformin improves hepatic lipid metabolism [19, 20]. In the non-pregnant state, metformin reverses fatty liver disease in obese leptin-deficient mice [21], blocks hepatic steatosis, liver inflammation, and fibrosis in a non-diabetic nonalcoholic steatohepatitis (NASH) mouse model [54], and counter-regulates diet-induced SREBP-1 and fatty acid synthase (FASN) protein expression in a mouse model [55]. Although maternal metformin administration reversed HCAL diet-induced SREBP-1 protein expression in maternal livers (Fig. 2), it had no effect on HCAL-induced hepatic (total) triglyceride-associated FA levels (Fig. 1a), fatty acid profile changes (Fig. 1b-e and Table 2) or FA-related gene expression in maternal livers (Table 3). In fact, metformin slightly increased total triglyceride-associated FA levels in the maternal rat liver, a finding that is consistent with the reported increase in circulating triglyceride levels and atherogenic plasma values in women with GDM who were treated with metformin vs. insulin later in pregnancy [56]. These results suggest potentially differential regulation of FA metabolism by metformin in the pregnant and non-pregnant states. Alternatively, in our study HCAL diet-induced changes in the maternal liver might not have been significant enough to be modified by metformin, a higher dose of metformin might be required in pregnancy, or the duration of metformin treatment was too short to observe measurable changes. However, this same HCAL diet regimen induced both GDM [25] and hyperinsulinemia [24] in pregnant rats.

Prenatal metformin exposure may affect the fetal compartment and lead to long-term programming effects on fetal metabolism [57], which may be positive. We and others have shown that maternal metformin exerts anti-inflammatory effects [24, 54, 58, 59], including the reduction of cytokines/chemokines in the fetal plasma and amniotic fluid in a rat model of diet-induced obesity/metabolic syndrome [24]. Although we did not observe significant HCAL-induced maternal liver-associated inflammation, elevated IFNγ concentrations were found in the fetal livers, and these were attenuated by maternal metformin administration (Fig. 6). Consistent with the fetal programming effects of metformin, *in utero* metformin exposure reversed diet-induced overall MUFA (Fig. 3c), as well as HCAL-diet reduced n3 FA (Fig. 3e), 18:1n9 (oleic acid) levels, and 22:6n3 (DHA) levels in fetal livers (Table 4). Although not significant, metformin slightly attenuated HCAL-induced *D5d* and *D9d* mRNA expression in the fetal livers (Table 3). By contrast, except for 22:5n6 (a minor phospholipid-fatty acid in the brain), *in utero* metformin exposure had no effects on diet-induced FA changes in the fetal brains (Table 5).

Our data show significant effects of maternal obesogenic-diet exposure and metformin treatment during metabolic syndrome in pregnancy on FA composition and FA metabolism in the fetal compartment. However, this study has several limitations. Methodologically, the high calorie diet was provided only 5 weeks prior to pregnancy and throughout pregnancy (3 weeks) and thus, represents ‘acute diet-induced obesity’. A longer HCAL diet-feeding period might better reflect the chronic obesity observed in humans. Because the normal and HCAL diets (± metformin) were provided *ad libitum* we were unable to assess exact intakes. Also, maternal metformin treatment was modeled after rodent studies performed in the absence of pregnancy. Perhaps higher doses of metformin are required during pregnancy. Finally, these findings are difficult to extrapolate to humans, which is true for all studies using laboratory animals. Nevertheless, this model may provide us with a better understanding of the pathophysiological changes observed with maternal obesity/metabolic syndrome and the use of metformin for obesity-related metabolic syndrome during pregnancy.

It is important to note that maternal metformin treatment in humans does not compromise neurodevelopmental outcomes when measured in two year old children [60, 61]. Our results show that metformin promotes preservation of fetal DHA (despite reduced maternal liver DHA) important for fetal/neonatal brain development. Recent studies by Salomaki and co-workers revealed that the fetal liver is an important target of maternal metformin and that protective effects on offspring were observed when dams were exposed to a high fat diet (vs. normal diet) prior to/during metformin treatment [57, 62]. Our study differs in that a high fat, high sugar diet was administered to dams (prior to/during metformin treatment) rather than a regular diet [57] or a high fat diet [62]; the high fat, high sugar diet was chosen to better reflect the Western diet. Although our study was not designed to investigate the effects of a maternal high calorie diet (±metformin) on long-term offspring outcomes, the data support the potential fetal programming effects of maternal obesity via changes in fetal fatty acid profiles and fetal liver IFNγ concentrations, with reversal by maternal metformin administration. Both GDM and adult obesity are increasing [3, 4, 63] and thus, represent widespread problems with potentially serious implications for mothers and their offspring. Therefore, future studies will focus on investigating the effects of metformin and other anti-diabetic and/or lipid normalizing strategies in the setting of obesity/metabolic syndrome during pregnancy on neonatal complications and long-term adverse metabolic consequences in the offspring. These studies would facilitate the development of interventions to mitigate the adverse effects of maternal obesity and metabolic syndrome on offspring health in the short- and long-terms.

## Conclusions

Obesogenic diet consumption by pregnant mice led to changes in the fatty acid composition of maternal and fetal livers, including reductions in healthy PUFAs and anti-inflammatory n3 FAs. *In utero* HCAL diet exposure increased liver IFNγ concentrations without affecting maternal liver IFNγ concentrations. While maternal metformin treatment did not significantly alter diet-induced maternal liver fatty acid changes, fetal exposure to metformin attenuated diet-induced changes in fetal liver DHA levels, anti-inflammatory n3FAs and liver IFNγ concentrations. Thus, maternal metformin might be beneficial for fetal/neonatal outcomes in the setting of maternal obesity and/or metabolic syndrome.

## Additional file

Additional file 1: Table S1. Cytokine and chemokine profiles in maternal and fetal livers following normal and high calorie diets (±metformin). (DOCX 21 kb)

## Abbreviations

AA: Arachidonic acid
DHA: Docosahexaenoic acid
FA: Fatty acid
GD: Gestational day
GDM: Gestational diabetes mellitus
HCAL: High calorie
MUFA: Monounsaturated fatty acid
PUFA: Polyunsaturated fatty acid
qPCR: Quantitative polymerase chain reaction
SFA: Saturated fatty acid
