# Supplementary material for: The effects of prenatal metformin on obesogenic diet-induced alterations in maternal and fetal fatty acid metabolism
Source: Nutr Metab (Lond). 2016 Aug 22;13(1):55. doi: 10.1186/s12986-016-0115-9 (PMC4994240; doi:10.1186/s12986-016-0115-9)
Supplement: Additional file 1: Table S1. — Cytokine and chemokine profiles in maternal and fetal livers following normal and high calorie diets (±metformin). (DOCX 21 kb) [file 12986_2016_115_MOESM1_ESM.docx]

Additional file 1: Table S1. Cytokine and chemokine profiles in maternal and fetal livers following normal and high calorie diets (±metformin).

| **Maternal Liver** | | | | | **Fetal Liver** | | |
| --- | --- | --- | --- | --- | --- | --- | --- |
|  | NORM | | HCAL | HCAL±MET | NORM | HCAL | HCAL±MET |
|  | pg/g | | pg/g | pg/g | pg/g | pg/g | pg/g |
| CXCL1 | | 11,692  (±12420) | 14,923  (±15,609) | 17,034  (±13498) | 27857  (±12396) | 28434  (±10877) | 21381  (±6330) |
| IFNγ | | 343  (±173) | 340  (±214) | 265  (±87) | 153  (±69) | 251  (±99)^A*^ | 163  (±53)^B*^ |
| IL-1β | | 9892  (±2855) | 10721  (±4988) | 8288  (±2785) | 39138  (±11545) | 51247  (±18505) | 34901  (±12035) |
| IL-4 | | 30  (±54) | 8  (±17) | 16  (±27) | 38  (±30) | 34  (±33) | 30  (±29) |
| IL-5 | | 5132  (±2117) | 5067  (±1429) | 5740  (±1894) | 647  (±829) | 965  (±921) | 644  (±632) |
| IL-6 | | 7055  (±2238) | 6304  (±1060) | 6314  (±1851) | 6657  (±1491) | 6906  (±1897) | 6771  (±1973) |
| IL-10 | | 328  (±499) | 155  (±101) | 245  (±198) | 290  (±142) | 397  (±148) | 332  (±147) |
| IL-13 | | 221  (±84) | 182  (±80) | 168  (±46) | 304  (±86) | 355  (±126) | 250  (±76) |
| TNFα | | 414  (±142) | 527  (±196) | 400  (±81) | 643  (±188) | 684  (±203) | 550  (±128) |
| NORM = normal diet, HCAL = high calorie diet, MET = metformin, SD = standard deviation  CXCL1 = CXC-motif ligand 1  A = NORM vs. HCAL, B = HCAL vs. HCAL+MET; *=p<0.05. Data are expressed as mean ± SD (pg/g). | | | | | | | |
